# Clinical efficiency of allergen-specific immunotherapy with fungal allergens in patients with chronic polypous rhinosinusitis against the background of fungal sensitization

**DOI:** 10.25122/jml-2021-0389

**Published:** 2022-03

**Authors:** Olesia Mykhailivna Herych, Vasyl Ivanovich Popovych, Ivanna Vasylivna Koshel, Diana Tadeivna Orishchak, Ostap Romanovych Orishchak, Yaroslav Romanovych Maksymenko, Andrii Volodymyrovich Bocharow, Petro Romanovych Herych

**Affiliations:** 1. Department of Otorhinolaryngology-Head and Neck Surgery, Ivano-Frankivsk National Medical University, Ivano-Frankivsk, Ukraine; 2. Department of Therapy and Family Medicine of Postgraduate Education, Ivano-Frankivsk National Medical University, Ivano-Frankivsk, Ukraine; 3. Department of Surgery, Bukovinian State Medical University, Chernivtsi, Ukraine; 4. Department of Internal Medicine No.1, Clinical Immunology and Allergology named after Academician E.M. Neiko, Ivano-Frankivsk National Medical University, Ivano-Frankivsk, Ukraine

**Keywords:** chronic polypous rhinosinusitis, fungal sensitization, specific immunotherapy, visual analog scale, FS – fungal sensitization, CPRS – chronic polypous rhinosinusitis, ASIT – allergen-specific immunotherapy, G1 – group 1, G2 – Group 2, VAS – visual analog scale

## Abstract

Fungal flora is one of the causes of inflammatory, including polypous, processes in the nasal cavity. In this regard, studies aimed at reducing the effect of fungal sensitization (FS) on the course of chronic polypous rhinosinusitis (CPRS) are relevant. The objective of the study was to evaluate the effect of various treatment options on the clinical course of the disease in patients with chronic polypous rhinosinusitis against the background of sensitization to fungi. The study included 90 patients with chronic polypous rhinosinusitis in combination with FS. The patients were divided into two groups – the first clinical group (G1) and the second clinical group (G2). G1 patients received allergen-specific immunotherapy (ASIT) according to the scheme. G2 patients received basic treatment. Evaluation of the clinical efficiency of ASIT was made based on complaints, assessment of symptom severity on a visual analog scale (VAS), and rhinoendoscopic examination. The treatment outcomes were evaluated on a 4-point scale, with excellent results (4 points) – complete remission of the disease during the follow-up period (6–12 months); good (3 points) – exacerbation of the disease 1-2 times a year, in mild form and removed by expectant treatment; satisfactory (2 points) – the number of exacerbations did not decrease. The use of ASIT therapy is pathogenetically justified and leads to a significant improvement in the clinical condition of patients with CPRS with FS.

## INTRODUCTION

Fungal flora plays a special role in the occurrence of polyposis. It is regarded as the root cause of the inflammatory, including polypous, process in the nasal cavity. Fungal sensitization is not always diagnosed in time and is considered an etiological factor of the disease, which is the reason for the insufficient effectiveness of treatment [1]. In most cases, the majority of patients show qualitative and quantitative changes of IgG4 and IgE in their blood, highlighting the importance of SPRS pathogenesis-related allergic reactions [2–4]. The process of sensitization most frequently occurs while inhaling fungal spores. Spores can exhibit their antigenic properties and cause a state of hypersensitivity when getting on the mucous membranes of the respiratory tract and conjunctiva. This process occurs without its further development and dissemination. Commonly, molds are associated with allergic pathology of the respiratory tract [5]. The development of mycogenic sensitization and allergies are determined by many factors: hereditary predisposition to allergic diseases, the dose of the allergen and the contact duration, the routes of allergen exposure [6]. According to studies conducted in 6 European countries, the identified fungal spores were: Cladosporium, Ascomycetes, Sporobolomyces, Basidiomycetes, Aspergillus, and Penicillium, yeast-like fungi [7, 8]. Most patients with existing sensitization to fungal allergens are diagnosed with chronic sinusitis (70%), and in nearly half of the cases, it is pyogenic polypous [9].

One of the most effective methods of treating allergic diseases is allergen-specific immunotherapy (ASIT) [10, 11]. The therapeutic effect of ASIT affects all stages of the allergic response, which is not typical for any pharmacological drugs. This treatment helps prolong the relapse-free interval, reduces the need for medications, prevents the expansion of the spectrum of allergens and the transition of mild forms to more severe ones [12–14].

In comparison with parenteral, non-invasive ASIT methods have a number of advantages: sufficiently high efficiency, the possibility of outpatient treatment, good tolerability, the ability to achieve high course doses of the allergen, a low risk of anaphylactic reactions, safety, and non-trauma [15–17].

The objective of the study was to evaluate the effect of various treatment options on the clinical course of the disease in patients with chronic polypous rhinosinusitis (CPRS) against the background of sensitization to fungi.

## MATERIAL AND METHODS

90 patients with polypous rhinosinusitis in combination with fungal sensitization (CS) were examined to describe the clinical course of CPRS. Patients were divided into two groups by blind randomized selection and uniformity group (G1 and G2). G1 patients (22 women and 14 men, aged 20–55) were treated with ASIT according to the scheme along with standard treatment. G2 patients (21 women and 33 men aged 20–55) received basic treatment for polypous rhinosinusitis following protocol No. 181 of March 24, 2009 (Protocol for Providing Medical Care to Patients with Chronic Sinusitis).

Indications for ASIT were hyperreactivity to fungi (positive allergic history data for FS), the presence of specific IgE antibodies to fungal allergens, positive skin tests with fungal allergens, and a high level of total IgE).

Allergic vaccination was made depending on the detected sensitization with an oral glycerin solution of the "H-AL mico per os" allergen extract – a mixture of external mold (Alternaria, Monilia, Botrytis, Cladosporium, Fusarium), or household mold mixture (Aspergillus, Penicillium, Rhizopus, Mucor).

Specific oral treatment was carried out in two phases: the first – cumulative or initial and the second – maintaining, in which the maximum tolerated dose was reached by gradually increasing the allergen concentration. The first phase duration was about 2.5–3 months. In the second phase, which lasted up to 6 months, the patient received the maximum allowable dose of the allergen to achieve a state of persistent hyposensitization. The therapeutic allergen was administered in the morning, 30 minutes before meals. The right amount of drops was dosed per teaspoon from the bottle with the allergen.

Treatment was initiated during the period of relative clinical remission after rehabilitation of infection foci. ASIT was performed against the background of basic anti-inflammatory treatment. The treatment efficiency was evaluated after 3, 6, and 12 months of treatment. Evaluation of the clinical effectiveness of ASIT was made based on complaints, assessment of the severity of symptoms, which included the subjective assessment of the overall severity in points (from 0 to 10). Based on the results of nasal endoscopy, the prevalence of the polypous process was assessed according to the recommendations of I. B. Soldatov (1997), based on which 4 levels of prevalence of the polypous process were distinguished: level I – polyps are not visualized; level II – polyps are visualized in the middle nasal passage; level III – polyps are visualized outside the middle nasal passages; level IV – polyps in the general nasal passage. The results of the treatment (ASIT) were evaluated on a 4-point scale, with excellent results (4 points) – complete remission of the disease during the follow-up period (6–12 months); good (3 points) – exacerbation of the disease 1–2 times a year, in mild form and removed by expectant treatment satisfactory (2 points) – the number of exacerbations did not decrease, but the overall state of health becomes much better than before immunotherapy; unsatisfactory (1 point) – treatment is discontinued due to lack of effect or on the introduction of an allergen, the patient constantly has worse symptoms.

## RESULTS

Carrying out a set of curative measures for patients with CPRS allowed stating certain levels of efficiency of the applied treatment regimens. Thus, after 3 months of treatment, patients in both groups showed positive dynamics of subjective sensations and clinical picture. The number of people with nasal breathing disorders in G1 was 58.33%, in G2 – 42.59% against 82.22% – before treatment. After 6 months, the number of people with nasal breathing disorders in G1 decreased to 25.00% compared to the start of treatment, which was significantly less than in the G2 group (29.62%) (p<0.05). Similar positive changes after the ASIT were recorded concerning complaints of olfactory disorders. Thus, after 3 months of treatment, the number of people with complaints of hyposmia in G1 was 86.11%, in G2 – 83.33% (against 93.33% – before treatment); after 6 months of treatment, the number of patients with complaints of hyposmia in G1 decreased to 41.67% (in G2 – 79.63%; p<0.05). The average value of the severity of symptoms on the VAS scale after 3 months of treatment in patients with G1 was 4.77±0.21 points versus 6.19±0.21 before treatment, and after 6 months – 3.29±0.22, which is 1.8 times less than before treatment and 1.4 times less than in patients from G2 who received basic treatment (p<0.05).

The results of nasal endoscopy showed that after treatment, polyps were not visualized in 5.88% of patients with G2 (basic treatment) and 22.22% – G1 (ASIT), which was 3.7 times more (p<0.05). Furthermore, stage IV prevalence of the polypous process after 6 months was diagnosed in 27.78% of the examined patients in G2 and only in 16.67% in G1 (p<0.05).

## DISCUSSION

Evaluation of treatment results on a 4-point scale after 6 months of ASIT showed positive treatment results in 85.7% of patients. Excellent and good results were observed in 78.6% of the examined patients, satisfactory – in 7.1% of patients. After 12 months of ASIT, excellent and good results were observed in 86.1% of patients, exacerbation occurred 1–2 times a year, and these were mild and removed by expectant treatment. In addition, there was an improvement in the subjective assessment symptoms severity by 1.06 times compared to the follow-up period of 6 months, and in the rhinoscopic picture (the number of G1 patients diagnosed with Stage III polypous process after 12 months decreased by 11.1%), and a decrease in the volume of basic treatment. Satisfactory treatment results were determined in 13.8% of patients whose number of exacerbations did not decrease, but their overall well-being became significantly better than before specific immunotherapy, which indicates a stable effect after treatment.

The problem of the treatment of polyposis rhinosinusitis today remains highly relevant. This is due to the prevalence rate and lack of radical, prognostic treatment methods available to doctors. Substantially all methods used for CPRS treatment today, both surgical and medical, give a significant percentage of exacerbation in a relatively short time. According to the literature, 55% of patients with chronic inflammatory diseases of the respiratory tract are sensitized to fungal allergens by mono- and polytype [18]. Up-to-date, allergen-specific immunotherapy (ASIT) is the most effective treatment method to reduce sensitization [19]. In our work, we used a variant of sublingual ASIT considered the most common and safe lately [20].

The therapeutic effect of ASIT affects all stages of the allergic response, which is not typical for any pharmacological drugs. Carrying out this therapy helps prolong the recurrence-free period, reduces the need for drugs, and prevents the expansion of the spectrum of allergens and the transition from mild to more severe forms of the disease. [21].

## CONCLUSION

ASIT treatment is pathogenetically justified and significantly improves the clinical condition and quality of life of patients with CPRS with FS. After 6 months of treatment in G1, the severity of clinical symptoms decreased by 1.8 times compared to the situation before treatment and by 1.4 times compared to G2 patients (p<0.05). The number of patients with complaints of hyposmia in G1 decreased to 41.67% compared to G2 – 79.63% (p<0.05). The severity of the polypous process in G1 patients was 1.6 times lower than in G2, which contributed to the positive clinical effect of treatment in 86.1% of patients. The conducted studies have shown high efficacy, good tolerability, and safety of the non-invasive method of treatment (ASIT) in CPRS patients with FS.

## ACKNOWLEDGMENTS

### Conflicts of interest

The authors declare that there is no conflict of interest.

### Ethical approval

The approval for this study was obtained from the Ethics Committee of the HSEEU Ivano-Frankivsk National Medical University, Ivano-Frankivsk, Ukraine (approval ID: 123/21, 21.09.2021).

### Consent to participate

Written informed consent was obtained from the patients.

### Data availability

Further data is available from the corresponding author on reasonable request.

### Funding

This study was funded by the Ivano-Frankivsk National Medical University.

### Authorship

OH contributed to conceptualizing the study. VP and IK contributed to the methodology. DO contributed to writing the original draft. OO contributed to editing the manuscript. YM contributed to data collection. AB contributed to data curation and PH contributed to data analysis.
